# Adolescent social emotional skills, resilience and behavioral problems during the COVID-19 pandemic: A longitudinal study in three European countries

**DOI:** 10.3389/fpsyt.2022.942692

**Published:** 2022-08-01

**Authors:** Baiba Martinsone, Ieva Stokenberga, Ilze Damberga, Inga Supe, Celeste Simões, Paula Lebre, Lúcia Canha, Margarida Santos, Anabela Caetano Santos, Ana Marta Fonseca, Dória Santos, Margarida Gaspar de Matos, Elisabetta Conte, Alessia Agliati, Valeria Cavioni, Sabina Gandellini, Ilaria Grazzani, Veronica Ornaghi, Liberato Camilleri

**Affiliations:** ^1^Department of Psychology, University of Latvia, Riga, Latvia; ^2^Department of Education, Social Sciences and Humanities, Faculty of Human Kinetics, University of Lisbon, Lisbon, Portugal; ^3^Environmental Health Institute, Faculty of Medicine, University of Lisbon, Lisbon, Portugal; ^4^Institute of Ethnomusicology (INET-MD), Faculty of Human Kinetics, University of Lisbon, Lisbon, Portugal; ^5^Social Adventure Association, Lisbon, Portugal; ^6^“Riccardo Massa” Department of Human Sciences for Education, University of Milano-Bicocca, Milan, Italy; ^7^Department of Statistics and Operations Research, University of Malta, Msida, Malta

**Keywords:** COVID-19, mental health, social emotional learning, behavioral problems, adolescents, multi-informant approach, longitudinal research

## Abstract

**Objectives:**

The consequences of long-lasting restrictions related to the COVID-19 pandemic have become a topical question in the latest research. The present study aims to analyze longitudinal changes in adolescents’ social emotional skills, resilience, and behavioral problems. Moreover, the study addresses the impact of adolescents’ social emotional learning on changes in their resilience and behavioral problems over the course of seven months of the pandemic.

**Methods:**

The Time 1 (T1) and Time 2 (T2) measuring points were in October 2020 and May 2021, characterized by high mortality rates and strict restrictions in Europe. For all three countries combined, 512 questionnaires were answered by both adolescents (aged 11-13 and 14-16 years) and their parents. The SSIS-SEL and SDQ student self-report and parent forms were used to evaluate adolescents’ social emotional skills and behavioral problems. The CD-RISC-10 scale was administered to adolescents to measure their self-reported resilience. Several multilevel models were fitted to investigate the changes in adolescents’ social emotional skills, resilience, and behavioral problems, controlling for age and gender. Correlation analysis was carried out to investigate how changes in the adolescents’ social emotional skills were associated with changes in their resilience and mental health adjustment.

**Results:**

Comparing T1 and T2 evaluations, adolescents claim they have more behavioral problems, have less social emotional skills, and are less prosocial than perceived by their parents, and this result applies across all countries and age groups. Both informants agree that COVID-19 had a negative impact, reporting an increment in the mean internalizing and externalizing difficulties scores and reductions in social emotional skills, prosocial behavior, and resilience scores. However, these changes are not very conspicuous, and most of them are not significant. Correlation analysis shows that changes in adolescents’ social emotional skills are negatively and significantly related to changes in internalized and externalized problems and positively and significantly related to changes in prosocial behavior and resilience. This implies that adolescents who experienced larger development in social emotional learning also experienced more increase in resilience and prosocial behavior and a decrease in difficulties.

**Conclusion:**

Due to its longitudinal design, sample size, and multi-informant approach, this study adds to a deeper understanding of the pandemic’s consequences on adolescents’ mental health.

## Introduction

In 2020 and 2021, the Coronavirus disease of 2019 (COVID-19) pandemic, whose causative agent is the SARS-CoV-2 virus, disrupted people’s lives worldwide. Although SARS-CoV-2 infections among children and adolescents cause less severe illness and fewer deaths than in adults, direct and indirect consequences of preventive measures against the virus were nonetheless felt by these groups. The measures adopted by governments, such as forced social lockdowns and the closure of public facilities to prevent the spread of the virus, caused multiple restrictions on human activities and physical interactions and a growing recognition of the effects on children and adolescents’ mental health (1–3).

Adolescence is a period of increased social and emotional development (4, 5). One of the major tasks in this period is the development of a cohesive personal identity. During the first years of adolescence, it is possible to see a significant self-understanding growth that sets the stage for critical elements of identity: self-awareness, the definition of own values, goals and future aspirations (6). Adolescence is also a crucial social expansion period, where the development of social cognitive skills is vital for healthy integration with others and in society (7). Relationships with social contexts show significant changes. Adolescents question parental authority and demand autonomy which can lead to conflicts (6). Parent-child conflicts during adolescence had been pointed out in the literature as normative. A meta-analysis on this scope also suggests that conflict and aggression in parent–child relationships negatively impact adolescent development (8). Nevertheless, Smetana and Rote (9) refer that these conflicts are temporary difficulties in parent-child relationships that help families redefine relationships from a more hierarchical to a more democratic dynamic. In this stage, adolescents’ peers and friends’ relationships also gain particular importance (8). The warmth, reciprocal understanding, and trustworthiness present in friendships are especially important for positive social and emotional development (6). Being with friends is one of adolescents’ favourite leisure activities (10). Nowadays, being with friends happens also through digital channels. The HBSC international report of 2018 (11) report that one in seven adolescents prefer to communicate online with their friends to discuss intimate matters.

Over the last two years, adolescents have been heavily confronted with social isolation, online learning, and routine disruptions for extended periods (12). Since the pandemic’s beginning, multiple studies regarding its impact on mental health have been published. Increased internalizing problems, such as anxiety and depression, and externalizing problems, such as anger, and reduced life satisfaction have been reported in school-aged children (1, 13–16). In addition, sleep problems, sadness, boredom, isolation, separation from peers, increased use of social media, reduced academic adjustment, and conflicts have been reported by other researchers (13, 15, 17–19). Systematic reviews have been consistent regarding adverse effects on adolescents’ well-being and mental health. These reviews also refer to a high prevalence of the COVID-19-related fear, fatigue, and distress in comparison with pre-pandemic estimates (20–23) and post-traumatic stress (22), with older adolescents and girls being more affected by these problems (2, 14, 16). Moreover, in a cross-cultural study with data from Italy, Spain and Portugal, including parents of 1,480 children from 3 to 18 years old, data showed an increased screen time and reduced physical activity (24).

Children and adolescents who experienced pre-pandemic vulnerabilities, namely lower socio-economic status, lower family support, and lower social emotional skills, experienced more significant mental health problems due to a reduction in the family income, problems with connectivity for online schooling, family conflicts, and neglect (16, 17, 20, 25, 26). Also, children and adolescents with neurodevelopmental and/or chronic physical conditions (3, 23) and youth living in rural areas were more likely to experience worsening mental health (27).

In the United Kingdom, the Co-SPACE Study (COVID-19: Supporting Parents, Adolescents and Children during Epidemics Study), which collected data monthly from over 9,000 parents/carers of 4-16-year-olds and over 1,300 adolescents (11-16-year-olds), found that participating children’s mental health worsened during lockdowns and school closures and improved as restrictions eased (28). In this study, it was also found that primary school-aged children (4-10-year-olds) were more likely than secondary school-aged children (11-16-year-olds) to have persistently poorer or worsening behavioral and concentration symptoms (29).

Nevertheless, positive outcomes were also identified. For example, online schooling that allowed the continuation of the learning process was associated with less academic and social stress (17). However, it is important to highlight that internet access is not equal, and those with connectivity issues, mainly from the poorest and remote areas, had their learning process compromised (16). Another example of a positive outcome is the decreased exposure to some risks at school, such as bullying, since children became more protected with the imposed restrictions and online classes (16). Being able to spend time with family members and having more time for homework and personal development were other positive outcomes pointed out by students (18). Along with this, the increased use of social media to maintain social contacts with peers and family members once children and adolescents were removed from their social contexts, like school (16), with the lockdown could also function as a protective factor.

To add to the pandemic outcomes discussion, a critical aspect in this scope is the variability in the pandemic’s effects (17, 30, 31). For example, Branje and Morris (17) report that despite its negative impact, many adolescents were able to face this adversity, and some even increased their social, emotional, or academic adjustment. In the same line, the study conducted by Salmela-Aro (31) found five different profiles of change in well-being, presenting the increasing well-being profiles as a growth in their intrapersonal social emotional competencies.

These results highlight the adolescents’ and their proximal systems’ ability to cope with this unexpected adversity. As Cefai et al. (32) mentioned, the pandemic presented an opportunity to obtain more insight into developing more resilient systems – in society, schools, and families – and find out which strategies contributed to their resilience and mental health and well-being using appropriate facilities, resources, and interventions (32). In this scope, previous evidence shows that social and emotional competencies are critical protective factors impacting the decrease in internalizing and externalizing problems and improvements in academic achievement (32).

During the pandemic, some studies reported that individual and family coping abilities and social support were significant predictors of positive mental health outcomes (33, 34). The available evidence identifies aspects of adolescents’ resilience to COVID-19, protecting against the mental health problems caused by the pandemic (34, 35). Some studies on aspects of adolescent resilience during the pandemic found that variables such as cognitive appraisal and humor (36) or task-oriented and avoidance-oriented coping styles (35) seem to protect against the mental health problems caused by the COVID-19 pandemic. A study conducted in Italy (37) found a significant positive association between social emotional skills and resilience skills. In turn, social emotional skills explained externalizing problems and prosocial behavior, and resilience skills explained internalizing and externalizing problems. Also, Deng et al. (38) found that emotion regulation strategies were effective for positive outcomes during the pandemic, but primarily for youth with lower COVID-19 stress-related factors.

Although these relevant data were gathered during recent times, many authors warned that caution is needed when interpreting the results and the importance of not generalizing since each country’s pandemic situation will influence how participants perceive themselves or others (13, 14, 39). Interestingly, transcultural and longitudinal data for Portugal, Spain, and Italy reported higher anxiety values in Spain and higher depression scores in Italy and Spain than in Portuguese children and adolescents (40). Another important aspect that further research needs to consider is the need to pay attention to the impact on different age groups since developmental timing is a critical variable in this context (41). The evidence in this scope presents other challenges since studies are mainly cross-sectional, retrospective, and longitudinal, comparing data collected before the pandemic (19, 31, 42, 43). Cross-sectional studies help to understand the immediate or short-term impact of the pandemic only at a particular time point, lacking insight into the long-term consequences (44). In addition, some longitudinal studies were carried out during the pandemic (40) but only for a short period (45, 46). Given the rapidly evolving nature of the COVID-19 pandemic and the methodological challenges involved with identifying its impact, interdisciplinary and longitudinal research cohorts conducting repeated assessments of mental health (ideally with baseline measures) have been referred to as a key to understanding the long-term impacts of the pandemic (47).

As far as the conceptual and methodological issues affecting the mental health assessment of children and adolescents are concerned, previous studies have drawn several recommendations. These recommendations are based on the understanding, identification, evaluation, and treatment of youngsters recovering from disaster contexts, such as the need to use standardized batteries and to focus on the cultural sensitivity of measures (48), as well as the value of using multi-informant data.

Before this specific pandemic period, a widely cited meta-analytic review had already stated that the level of agreement across studies on internalizing and externalizing symptomatology as reported by children, parents, mental health workers, teachers, and peers should be considered (49). This need is based on the fact that correlations between similar types of informants (e.g., mother, father) are usually higher than correlations between different types of informants (e.g., parents, teachers) or self-other correlations and that all are commonly in a low-to-moderate range. Given the low level of agreement, several investigators have recommended using multisource and multimethod data to assess children and adolescents [e.g., (49–51)]. More recently, the importance of multisource information so that it is possible to minimize constrictions arising from a single self-report assessment has been underlined (52).

The challenge of determining whether the result is about the construct itself or the evaluation method that may condition the question has also been reported (53). Particularly at the level of self-report instruments, the authors reinforce the difficulty of discerning issues such as vulnerability to faking, responding in a socially more desirable way, or ecological validity.

Although research during the COVID-19 pandemic with adolescents relied mainly on online self-report data (44), studies including pre-adolescents focused mostly on parents’ reports [for a meta-analysis, see (54)], with these reiterating the data obtained through self-reports. Regarding to self-report measures, namely the Strengths and Difficulties Questionnaire (SDQ), some authors state that caution is needed when assessing the mental health of children and adolescents. Although this measure shows validity and reliability (55, 56), it requires some caution as far as its suitability for cross-country comparisons is concerned due to the lack of a common acceptable model across countries, that is, its dimensional invariance (57).

As such, it seems that the need for identifying reliable data on changes in different adolescents’ mental health variables during the pandemic and the latter’s relationship with social emotional and resilience skills and behavioral problems is evident to provide evidence-based conclusions. Studies published on mental health indicators during the pandemic focus more on younger ages and more clinical perspectives, and evidence on the dynamic of resilience and the possible protective role of social emotional skills is very limited. Therefore, the present study aims to add to the existing evidence by addressing some of the gaps mentioned in the literature, namely the need for longitudinal studies with multi-informants, inclusion of older adolescents and addressing a positive perspective, namely resilience and social emotional skills.

Therefore, we posed two research questions for this study:

(1). What changes were there in adolescents’ self-reported and parents’ reported social emotional skills, resilience, and behavioral problems among age and gender groups in three European countries during the COVID-19 pandemic?

(2). Did social emotional learning relate to adolescents’ resilience and behavioral problems during the COVID-19 pandemic?

## Method

### Research context

This research is part of the Erasmus + Key Action 3 funded international project “Promoting mental health at schools” (PROMEHS). The project’s goal is to develop, implement, and evaluate an evidence-based universal curriculum focused on students’ and teachers’ mental health and propose recommendations for innovative educational policies. In total, seven European countries were involved in the project: Latvia, Italy, Portugal, Croatia, Romania, Greece, and Malta. The PROMEHS program included activities for students aged from 3 to 18 years to promote their social emotional learning and resilience, as well as to prevent social, emotional, and behavioral difficulties. To evaluate the efficacy of the program, a quasi-experimental study design was applied. Four age groups of students from preschool to high school (3-6, 8-10, 11-13, and 14-16 years) were selected and randomly divided into experimental and waiting-list control groups. Data were collected from three sources – students (with the exception of the preschool group), their parents, and teachers. The PROMEHS program was implemented and tested in the 2020/21 school year, which coincided with the time of the COVID-19 pandemic, affecting almost every aspect of life globally. It provided the opportunity to obtain longitudinal data from two measure points (T1 and T2) within the control group, which did not receive any intervention during the school year. Data from the control group of 11-16-year-old students and their respective parents from Latvia, Italy, and Portugal were used for the current study to test the dynamic of adolescents’ social emotional learning, resilience, and behavioral problems during the pandemic.

A longitudinal research design was used, following 512 adolescents through the 2020/21 school year. The selected period was characterized by strict social distancing measures, including remote learning due to no vaccinations for this age group being available, allowing us to evaluate the dynamic of the mental health indicators among adolescents going through this very tense phase of the pandemic. This broadens evidence published so far, which is mainly based on cross-sectional or retrospective designs or where pre-test data were collected before the beginning of the pandemic.

Additionally, we used a multi-informant approach, including both students’ and parents’ self-reports, to evaluate broad indicators related to mental health – namely social emotional learning, resilience, and behavioral problems – in three European countries. This provides a more reliable and valid estimation considering internal and external difficulties and important resources for adolescents’ healthy development and mental health.

### National regulations due to the COVID-19 pandemic during the 2020/21 school year in the three countries

This study took place in one of the waves of the COVID-19 pandemic in Europe (see Table 1), specifically characterized by different types of restrictions (see Table 2) (e.g., remote learning, prohibited gatherings, or even lockdown), high mortality rates due to the high prevalence of infection, and increasing but insufficient vaccination coverage.

**TABLE 1:** Daily new confirmed COVID-19 cases per million people, 7-day rolling average/cumulative confirmed COVID-19 deaths per million people 2020/2021 in Italy, Latvia, and Portugal.

Time point	Italy	Latvia	Portugal
1 November 2020	434.37/643.16	111.57/39.64	360.45/250.20
1 January 2021	239.06/1,236.11	474.04/344.95	405.29/685.69
1 March 2021	282.81/1,622.48	356.96/868.27	96.69/1,608.10
1 May 2021	203.75/2,004.94	337.83/1,145.73	41.91/1,669.56

Data source: COVID-19 Data Repository by the Center for Systems Science and Engineering (CSSE) at Johns Hopkins University.

**TABLE 2:** Policy responses to COVID-19 (number of days with certain policy) and vaccination rates (% of population partly/fully vaccinated) between 1 November 2020 and 1 May 2021 in Italy, Latvia, and Portugal.

Policy/Time point	Italy	Latvia	Portugal
School policy (remote learning at home): partly/fully	131/50	181/0	35/57
Workplace policy (closing or work from home): partly/fully	66/115	130/51	127/54
Stay-at-home policy	181	0	152
**Partly/Fully vaccinated (% of population)**			
1 January 2021	0/0.33	0/0.05	0/0.13
1 March 2021	2.4/2.7	0.94/1.6	2.9/3.5
1 May 2021	10/14	3/12	9.3/17

Data source: Official data collated by Our World in Data; Oxford COVID-19 Government Response Tracker, Blavatnik School of Government, University of Oxford.

Notes.

School policy: Partly - Policy requires on-site school closing only at some levels or categories, e.g., just high school.

Fully - Policy requires on-site school closing at all levels.

Workplace policy:

Partly - Policy requires closing or work from home for some sectors or categories of workers.

Fully - Policy requires closing or work from home all but essential workplaces (e.g., grocery stores, doctors).

Stay at home policy:

Policy requires not leaving house with exceptions for daily exercise, grocery shopping, and “essential” trips.

#### Latvia

The prevalence of COVID-19 infections rapidly increased between October and December 2020 and remained relatively stable until May 2021. Considering the low infection rates in the first wave of the pandemic, Latvia started with a relatively low number of cumulative confirmed COVID-19 deaths on 1 November 2020 (39.64 per million people), but there was a rapid increase, and 1,145.73 deaths per million were reached on 1 May. Vaccinations started on 28 December 2020, but on 1 May, only 15% of the population had received at least one dose, and only 3% were fully vaccinated. At the same time in Portugal, 25% had received at least one dose and 9% were fully vaccinated, while in Italy, these figures were 24 and 10%, respectively. There was no vaccination available for adolescents in Latvia (similar to Italy) until June 2021.

In Latvia, there was a state of emergency due to the COVID-19 pandemic from 9 November 2020 to 7 April 2021, and a distancing policy was in place. Children (aged 10 and younger) continued on-site learning until January 2021, whereas remote learning was introduced for older students (in December 2020 for students aged 11-13, and in October 2020 for students 14 and above). Remote learning continued mostly in all age groups until the end of the school year in May 2021. There was individual support available on-site for students who needed assistance from February 2021, and in some regions with lower infection prevalence, some of the older students were able to return to some activities at school on-site from April 2021. Overall, almost all the adolescents learned remotely. Classes were organized according to curricula; at least 30% of them ought to have been online interaction, and the remaining time was used for independent learning and individual tasks. Extracurricular activities were implemented remotely or individually on-site, and outdoor activities (e.g., sports training) were allowed in groups of up to 10 students.

Parents were encouraged to work from home whenever it was possible to carry out duties remotely, and employers were instructed by the government to actively encourage and support teleworking. Gatherings outside the household were prohibited, and a curfew during which individuals had to stay at their place of residence between 22:00 and 05:00 except for emergencies and work-related matters was introduced during the holidays (from 30 December 2020 to 4 January 2021) and weekends (from 8 January to 7 February 2021).

#### Italy

Italy was heavily hit by the pandemic from its beginning. Despite the strong health system, it was the hardest-hit European country for months, setting records in terms of cases and deaths (58). The Italian government declared a state of emergency from January 2020, then prolonged it until the end of March 2022. This challenging situation negatively affected both parents and students of every school grade (40) because the negative feelings (e.g., fear, helplessness, etc.) triggered by the pandemic were combined with nationwide lockdowns and the obligation of distance learning, which was protracted until the end of the 2019/20 school year.

In autumn 2020, contagions precipitously increased due to the COVID-19 variants. Thus, in October 2020, the government set curfews and strict rules concerning leisure and social activities, toughening rules on social distancing and home confinement. Based on contagion percentages, Italian regions were divided into red, orange, and yellow zones. Distance learning was required for all high schools, as well as second and third grades of middle schools located in red zones.

After a winter holiday break during which there were restrictions, in January 2021, middle and high secondary schools located in non-red zones gradually started to provide on-site learning, with between 50-75% of the students being present in the classroom and the remaining continuing distance learning. When on-site, masks and social distancing were compulsory. Nevertheless, single classrooms or whole schools often experienced forced closures due to students and/or teachers testing positive for COVID-19. At a national level, movements across regions were prohibited, with the exception of those related to work and health reasons.

This situation continued until April 2021, when the government imposed a national lockdown on the occasion of the Easter holidays to limit the spread of the contagion. Access to schools depended on the seriousness of the contagion in the specific zone and the school’s grade: in red zones, middle (with the exception of the first grade) and high schools kept on providing distance learning; in orange and yellow zones, middle schools provided on-site learning, while high schools adopted the 50-75% learning approach. Since then, control measures have been progressively loosened, in parallel with the successful vaccination campaign and a decrease in cases.

#### Portugal

The first COVID-19 case in Portugal was confirmed on 2 March 2020, and the first pediatric case on 7 March 2020 (59). The pandemic in Portugal forced two lockdowns, one in March 2020, when the World Health Organization declared a pandemic situation, and another in January 2021. Regarding health, a huge level of hospital demands were observed due to the increased number of cases with severe illness and required hospitalization caused by COVID-19 infections and increased stress on health services was observed. According to the Direção-Geral da Saúde (59), the highest rate of deaths due to a COVID-19 infection (200 per day) occurred in January 2021. Nonetheless, children and adolescents presented a lower risk of developing a severe form of the disease and required less hospital care (60).

These confinements caused significant changes in the functioning of schools. The majority were rapidly modernized for online learning provision, taking into account the moment’s needs. Although there was a shortage of online education resources and a lack of a culture of autonomous work among the students, most teachers, students, and families organized themselves relatively efficiently to adapt to this period of mandatory physical distancing, social confinement, and school closure. However, Portuguese society’s social and economic inequalities became an obstacle to education. Some children could not follow the classes, missing curricular objectives. However, an effort was made to equip students from lower social and economic groups with the necessary technology so as to cover a large part of this population during the confinement periods.

The creation of a support website, digital terrestrial television classes, the creation of five channels on YouTube, and distance learning online training in schools using the MOOC (Massive Open Online Course) format occurred between February and July 2021, and there was also the implementation of digital textbooks (PDT) and the production of digital educational resources (61).

## Participants

The sample of 512 adolescents consisted of 245 boys with a mean age of 13.16 years and a standard deviation of 1.67 years and 267 girls with a mean age of 13.12 years and a standard deviation of 1.76 years. The mean age and standard deviations were calculated by taking their average age between T1 and T2. The participants were selected from Italy (*n* = 102, 19.9%), Latvia (*n* = 284, 55.5%), and Portugal (*n* = 126, 24.6%) and were stratified by gender and age group (11-13 and 14-16 years). The mean ages of Italian, Latvian, and Portuguese boys (13.27, 13.17, and 13.03 years, respectively) and girls (13.21, 13.03, and 13.12 years, respectively) varied marginally by country and gender.

Table 3 represents the demographic characteristics of both the national samples and the whole sample of the study.

**TABLE 3:** Characteristics of the sample.

		Country			Total
		Italy	Latvia	Portugal	
Gender	Boys	53(10.3%)	136 (26.6%)	56 (10.9%)	245 (47.8%)
	Girls	49 (9.6%)	148 (28.9%)	70 (13.7%)	267 (52.2%)
Age-Group	11-13 years	52 (10.2%)	148 (28.9%)	55 (10.7%)	255 (49.8%)
	14-16 years	50 (9.8%)	136 (26.6%)	71 (13.9%)	257 (50.2%)

### Procedure

**FIGURE 1:**
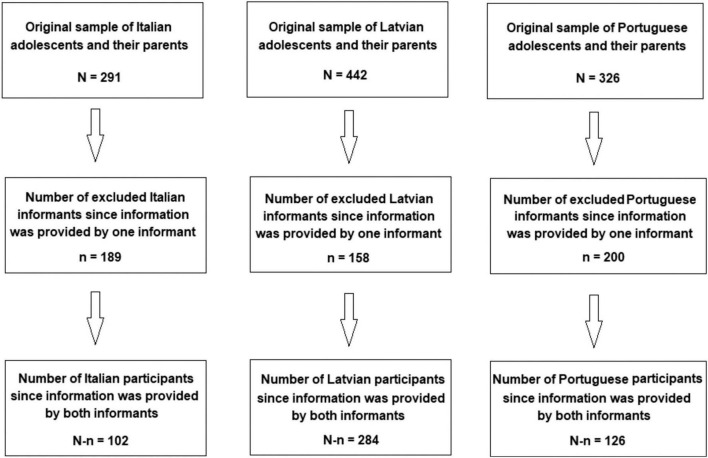
Flowchart of enrollment in each country.

The process of data collection took place twice: at the beginning and at the end of the 2020/21school year in October 2020 and May 2021, respectively. To ensure the participants’ privacy and to match T1 and T2 data, every participant received a unique anonymized code to be used when filling in the survey. Parents were assigned the same code as their children to enable comparisons between the two time points. Only adolescents and their parents who completed their respective questionnaires in both phases were included in the study. Figure 1 displays the flowchart of enrollement of participants in each country. The initial sample of 1,059 adolescents and their parents was reduced to 512 either because one informant or both informants did not provide the requested information. Nevertheless, a sample of 512 participants still guaranteed a maximum margin of error of 4.3%, assuming a 95% confidence level. Moreover, when the responses of single informants were added to data set the changes in the mean pre- and mean post-test scores were insignificant. This indicates that dropouts occurred across the whole range of social emotional skills, behavioral problems and resilience scales and had no impact on the findings.

In Latvia, researchers organized informative campaigns in eight schools in the Sigulda region. This region is a project partner, so all schools in the region were invited to participate. Initially, agreements with school principals and teachers regarding participation in the project were reached. Then informative letters with an invitation to participate in the survey were sent to parents. Parent surveys with informed consent placed on the main page were put into envelopes, coded, and delivered to every school by researchers. The envelopes were distributed among parents by a class teacher. Parents were asked to sign the informed consent, fill in the survey, and return them in sealed envelopes to the class teacher. The sealed envelopes were collected by the researchers, who opened them and checked if the informed consent of the parent had been given. Then the researchers attended the school and invited those adolescents with signed parental informed consent to take part in the survey. Before students completed the survey, they also gave their own written informed consent to participate. Students filled in their self-report surveys in paper form at school, in the presence of the researcher, who answered questions and collected all completed surveys immediately. Finally, the researchers input all the answers into an electronic data file. Only surveys of students who had, together with their parents, given their informed consent were included in the research. The Ethics Committee for Humanities and Social Sciences Research Involving Human Participants of the University of Latvia granted permission for the research on 12 December 2019.

In Italy, researchers contacted schools located in northern Italy, namely in the Lombardy and Piedmont regions, explaining the objectives and methodology of the project. After exploring teachers’ willingness to be involved in the research, school principals were asked to sign an agreement. All participants completed the survey online, with the exception of those parents who explicitly required it in a paper version. At the beginning of the survey, parents were required to provide their informed consent for themselves and their children. Before students completed the survey, they were also asked to give their informed consent to participate. The research was approved by the Ethical Committee of the University of Milano-Bicocca on 21 July 2020.

In Portugal, researchers organized meetings with local policymakers and wellness observatory organizations to present the project to them. Then those organizations contacted school principals who might be interested in participating in the project. Each school principal subsequently contacted and selected teachers interested in being part of the project. Meetings with principals, teachers, and school psychologists were held to present the project and the evaluation procedure. The researchers sent informed consent forms to the teachers, who then sent them on to the parents. After the teachers collected the parents’ consent form, they sent them a link to the online survey or, in some cases, they sent a paper version. Students completed the self-report forms in class. However, some students did theirs at home due to the distancing restrictions. An assent form was also given to the students. Students gave their consent in the online survey or, in some cases, in the paper version. When paper versions were used, they were sent by the teachers to the researchers, who input the data into the database. The Ethics Committee of the Environmental Health Institute at the University of Lisbon approved the research on 20 March 2020.

### Measures

Both the adolescents and their parents participating in the study were asked to complete the Strengths and Difficulties Questionnaire (SDQ) and the Social Skills Improvement System Social-Emotional Learning Brief Scales (SSIS-SEL), while the adolescents were also asked to complete the Connor-Davidson Brief Resilience Scale (CD-RISC-10).

The SDQ (62, 63) is a widely used tool to measure the mental health of children and adolescents. It consists of 25 items (5 per scale), allowing the researcher to evaluate difficulties in four areas of difficulty (emotional, conduct, hyperactivity, and peer problems) and one strength (prosocial behavior). In this questionnaire, a 3-point Likert-type scale ranging from 0 (not true) to 2 (certainly true) is used, and higher scores indicate more social, emotional, and behavioral difficulties or a greater intention to help others, respectively. The model with a summed score for internalized and externalized difficulties, as well as for total difficulty and prosocial behavior, was used in the current study.

The SSIS-SEL (64, 65) evaluates students’ social emotional learning. The measure consists of 20 items that allow one to estimate five domains of social emotional skills: self-awareness, self-management, social awareness, relationship skills, and responsible decision-making. Each item is measured on a 4-point Likert-type scale ranging from 0 (never) to 3 (almost always), and higher scores correspond to greater social emotional competence.

The CD-RISC-10 (66) is a self-report measure used to explore resilience among adolescents and adults. The short version of the scale (67) consists of 10 items on a 5-point Likert scale, from 0 (not true at all) to 4 (true nearly all the time), where higher scores indicate a greater ability to handle stress and to be more resilient.

The SDQ parent form was previously validated in the Latvian, Italian, and Portuguese languages. The Latvian SDQ self-report form and CD-RISC-10 were translated according to recommendations in the literature (68), namely translation, review, back-translation, review by experts, piloting in the target group, and final agreement among the experts’ committee to reach appropriate cultural, semantic, and conceptual equivalence with the original measure. The same procedure was applied to the Brief SSIS-SEL scales for their Latvian, Italian, and Portuguese versions (see Table 4).

**TABLE 4:** Reliability of SDQ, SSIS-SEL, and CD-RISC-10 scales (Cronbach’s alpha).

Subscale Informant		Country			Whole Group
		Italy	Latvia	Portugal	
Internalized difficulty	Child self-report	0.724	0.750	0.722	0.732
	Parent report	0.740	0.720	0.716	0.717
Externalized difficulty	Child self-report	0.702	0.701	0.751	0.712
	Parent report	0.745	0.781	0.784	0.777
Total difficulty	Child self-report	0.796	0.786	0.809	0.793
	Parent report	0.797	0.796	0.805	0.800
Prosocial behavior	Child self-report	0.607	0.668	0.664	0.656
	Parent report	0.675	0.663	0.649	0.672
Self-awareness	Child self-report	0.543	0.545	0.593	0.562
	Parent report	0.617	0.637	0.667	0.641
Self-management	Child self-report	0.586	0.585	0.648	0.619
	Parent report	0.693	0.705	0.686	0.702
Social awareness	Child self-report	0.743	0.710	0.721	0.733
	Parent report	0.785	0.819	0.771	0.804
Relationship skills	Child self-report	0.539	0.530	0.583	0.549
	Parent report	0.675	0.681	0.661	0.678
Responsible decision making	Child self-report	0.643	0.567	0.667	0.618
	Parent report	0.759	0.761	0.771	0.768
Social emotional learning	Child self-report	0.842	0.844	0.871	0.857
	Parent report	0.917	0.902	0.880	0.903
Resilience	Child self-report	0.840	0.839	0.845	0.843
	Parent report				

The Cronbach’s alpha coefficient was computed to assess the internal consistency of the items measuring each scale, where values exceeding 0.7 indicate good internal consistency and values ranging from 0.5 to 0.7 indicate acceptable internal consistency. The SSIS-SEL summed score reached good internal consistency in both parent and child self-reports, and SDQ difficulty scales were characterized as good in terms of their reliability in both parent and child self-report forms. The reliability of the prosocial behavior scale was acceptable in both parent and child self-report forms. Similarly, four out of the five SSIS-SEL scales in the child self-report forms reached an acceptable level of consistency; only social awareness reached a good level of consistency. This was similar to the parent forms, where only social awareness and responsible decision-making could be characterized as having good reliability in all languages.

### Data analysis

Descriptive statistics tables were generated to display the mean subscale scores for the SDQ, SSIS-SEL, and CD-RISC-10 questionnaires, where adolescents’ and parents’ evaluations were grouped by the adolescents’ gender, age, and country of residence. The Wilcoxon signed-rank test was used to determine whether mean scores in behavioral problems, social emotional skills, and resilience varied significantly between October 2020 and May 2021. A limitation of the Wilcoxon signed-rank test is that it does not investigate the impact of other explanatory variables on behavioral problems, social emotional skills, and resilience. To address this limitation, several multilevel models were fitted, using both the adolescents’ and parents’ evaluations, to determine whether the changes in social emotional skills, resilience, and behavioral problems between October 2020 and May 2021 varied significantly between gender and age-groups. Multilevel models are hierarchical linear mixed models and were used to accommodate the nested structure of the data, where adolescents are nested in schools, which in turn are nested in countries. Besides identifying the significant explanatory variables, multilevel models also measure the variance at each level of nesting. Finally, correlation analysis was used to investigate the strength of the relationships between changes in SSIS-SEL and changes in SDQ and CD-RISC-10. These analyses were carried out for each country separately and for all countries combined.

## Results

### Changes in adolescents’ social emotional skills, resilience, and behavioral problems during the COVID-19 pandemic

One way to measure changes in social emotional skills, behavioral problems and resilience scale scores is by computing the difference between the pre- and post-test scores and using this change score as the dependent variable. In this part of analysis, pre- and post-test scores were used rather than change scores to compare the parents’ perceptions with perceptions of adolescents on their social emotional skills, behavioral problems and prosocial behavior. Table 5 displays the mean scores of adolescents’ self-reported evaluations in behavioral problems, social emotional skills, and resilience in October 2020 and May 2021. These scores were calculated by clustering the adolescents by age (11-13 years, 14-16 years), gender (boys, girls), and country (Italy, Latvia, Portugal).

**TABLE 5:** Mean SDQ, SSIS-SEL, and CD-RISC-10 scores as reported by adolescents (*n* = 512).

Subscale	Age	Gender	Italy				Latvia				Portugal				Whole Group			
			2020		2021		2020		2021		2020		2021		2020		2021	
			Mean	SD	Mean	SD	Mean	SD	Mean	SD	Mean	SD	Mean	SD	Mean	SD	Mean	SD
Internalizing difficulty	11-13	Male	1.50	0.410	1.51	0.411	1.58	0.355	1.64	0.340	1.46	0.225	1.46	0.247	1.53	0.332	1.57	0.337
		Female	1.50	0.439	1.54	0.352	1.74	0.362	1.80	0.398	1.62	0.294	1.71	0.372	1.67	0.360	1.74	0.386
	14-16	Male	1.70	0.314	1.83	0.322	1.58	0.357	1.62	0.320	1.53	0.276	1.52	0.327	1.59	0.325	1.64	0.317
		Female	1.81	0.406	1.75	0.213	1.77	0.328	1.80	0.382	1.73	0.363	1.75	0.369	1.76	0.361	1.77	0.368
Externalizing difficulty	11-13	Male	1.47	0.267	1.60	0.310	1.68	0.307	1.63	0.326	1.55	0.345	1.63	0.326	1.60	0.315	1.62	0.319
		Female	1.47	0.340	1.39	0.276	1.60	0.267	1.68	0.321	1.56	0.377	1.57	0.410	1.57	0.316	1.61	0.348
	14-16	Male	1.65	0.201	1.63	0.396	1.64	0.245	1.69	0.298	1.62	0.296	1.66	0.406	1.64	0.278	1.67	0.332
		Female	1.58	0.232	1.55	0.262	1.55	0.271	1.62	0.297	1.67	0.360	1.61	0.373	1.59	0.345	1.60	0.335
Total difficulty	11-13	Male	1.48	0.301	1.55	0.309	1.63	0.274	1.64	0.289	1.51	0.218	1.54	0.245	1.57	0.270	1.60	0.281
		Female	1.48	0.302	1.46	0.272	1.67	0.268	1.74	0.314	1.59	0.306	1.64	0.332	1.62	0.291	1.67	0.324
	14-16	Male	1.67	0.172	1.73	0.265	1.61	0.237	1.66	0.242	1.57	0.254	1.59	0.342	1.61	0.253	1.65	0.280
		Female	1.69	0.308	1.65	0.209	1.66	0.256	1.71	0.301	1.70	0.286	1.68	0.315	1.68	0.291	1.69	0.295
Prosocial behavior	11-13	Male	2.52	0.237	2.43	0.327	2.42	0.343	2.41	0.388	2.64	0.286	2.52	0.352	2.49	0.354	2.44	0.386
		Female	2.57	0.352	2.61	0.329	2.55	0.317	2.42	0.356	2.65	0.307	2.69	0.274	2.57	0.322	2.51	0.365
	14-16	Male	2.55	0.314	2.30	0.283	2.27	0.435	2.33	0.404	2.57	0.371	2.53	0.288	2.39	0.371	2.37	0.386
		Female	2.59	0.346	2.39	0.241	2.52	0.275	2.54	0.311	2.62	0.393	2.64	0.377	2.56	0.361	2.54	0.367
Self-awareness	11-13	Male	2.80	0.432	2.69	0.520	2.95	0.436	3.00	0.426	3.22	0.465	3.15	0.419	2.97	0.482	2.96	0.481
		Female	2.85	0.566	2.84	0.571	3.12	0.462	3.01	0.519	3.26	0.586	3.12	0.543	3.10	0.520	3.00	0.519
	14-16	Male	2.66	0.529	2.68	0.243	2.82	0.407	2.83	0.523	3.06	0.426	3.04	0.369	2.85	0.447	2.85	0.455
		Female	2.79	0.528	2.73	0.420	2.99	0.459	2.91	0.444	2.95	0.394	2.95	0.455	2.94	0.433	2.89	0.461
Self-management	11-13	Male	2.62	0.489	2.33	0.532	2.89	0.478	2.82	0.485	3.06	0.545	2.87	0.551	2.86	0.525	2.71	0.516
		Female	2.61	0.530	2.77	0.460	3.00	0.509	2.90	0.512	2.91	0.662	2.95	0.584	2.91	0.579	2.89	0.534
	14-16	Male	2.60	0.608	2.49	0.332	2.81	0.450	2.84	0.464	2.88	0.568	2.79	0.381	2.78	0.512	2.76	0.505
		Female	2.56	0.420	2.75	0.207	2.91	0.500	2.94	0.410	2.88	0.406	2.81	0.557	2.83	0.504	2.87	0.538
Social awareness	11-13	Male	3.17	0.557	3.10	0.553	2.80	0.550	2.84	0.553	3.19	0.526	3.05	0.654	2.97	0.575	2.95	0.600
		Female	3.33	0.465	3.31	0.425	3.26	0.605	3.09	0.575	3.48	0.500	3.52	0.360	3.32	0.552	3.22	0.543
	14-16	Male	3.04	0.404	3.24	0.404	2.72	0.573	2.76	0.577	3.28	0.466	3.33	0.437	2.92	0.557	2.99	0.590
		Female	3.37	0.507	3.47	0.414	3.12	0.562	3.12	0.434	3.43	0.424	3.44	0.466	3.27	0.548	3.29	0.526
Relationship skills	11-13	Male	3.22	0.430	3.10	0.415	3.18	0.471	3.13	0.424	3.42	0.425	3.38	0.384	3.24	0.481	3.18	0.436
		Female	3.20	0.536	3.20	0.448	3.29	0.475	3.15	0.516	3.38	0.594	3.35	0.564	3.29	0.498	3.20	0.491
	14-16	Male	3.24	0.321	3.15	0.346	3.16	0.462	3.10	0.447	3.36	0.412	3.38	0.375	3.22	0.437	3.18	0.433
		Female	3.13	0.451	3.13	0.127	3.30	0.383	3.17	0.353	3.40	0.371	3.31	0.400	3.30	0.416	3.21	0.411
Responsible decision making	11-13	Male	2.87	0.404	2.91	0.600	3.09	0.505	3.09	0.470	3.18	0.545	3.14	0.521	3.06	0.504	3.06	0.528
		Female	3.09	0.482	2.88	0.610	3.23	0.467	3.23	0.457	3.42	0.519	3.39	0.543	3.25	0.480	3.20	0.539
	14-16	Male	2.97	0.671	2.76	0.707	3.00	0.440	3.01	0.491	3.34	0.422	3.28	0.405	3.08	0.510	3.03	0.522
		Female	3.24	0.502	3.05	0.351	3.18	0.357	3.21	0.338	3.44	0.386	3.40	0.401	3.27	0.434	3.24	0.454
Social emotional learning	11-13	Male	2.94	0.240	2.83	0.332	2.98	0.358	2.97	0.364	3.21	0.324	3.12	0.405	3.02	0.374	2.97	0.387
		Female	3.02	0.323	3.00	0.334	3.18	0.386	3.07	0.414	3.29	0.473	3.27	0.432	3.18	0.399	3.10	0.407
	14-16	Male	2.90	0.415	2.87	0.317	2.90	0.350	2.91	0.372	3.18	0.317	3.16	0.296	2.97	0.367	2.96	0.384
		Female	3.02	0.314	3.03	0.213	3.10	0.300	3.07	0.246	3.22	0.288	3.18	0.363	3.12	0.327	3.10	0.366
Resilience	11-13	Male	3.22	0.573	3.20	0.693	3.60	0.676	3.54	0.609	3.61	0.617	3.68	0.783	3.51	0.686	3.49	0.682
		Female	3.37	0.815	3.21	0.795	3.28	0.675	3.19	0.699	3.40	0.756	3.45	0.620	3.32	0.735	3.25	0.774
	14-16	Male	3.16	0.555	2.68	0.605	3.66	0.533	3.58	0.643	3.75	0.648	3.78	0.617	3.59	0.613	3.45	0.649
		Female	2.93	0.510	2.87	0.541	3.49	0.686	3.39	0.759	3.31	0.655	3.34	0.879	3.32	0.736	3.27	0.827

The parents’ reported changes in their adolescent children’s behavioral problems, social emotional skills, and resilience in the two phases are in Table 6.

**TABLE 6:** Mean SDQ, SSIS-SEL, and CD-RISC-10 scores as reported by parents (*n* = 512).

Subscale	Age	Gender	Italy				Latvia				Portugal				Whole Group			
			2020		2021		2020		2021		2020		2021		2020		2021	
			Mean	SD	Mean	SD	Mean	SD	Mean	SD	Mean	SD	Mean	SD	Mean	SD	Mean	SD
Internalizing difficulty	11-13	Male	1.37	0.291	1.43	0.327	1.45	0.296	1.45	0.335	1.34	0.234	1.34	0.197	1.40	0.284	1.42	0.309
		Female	1.25	0.463	1.43	0.286	1.48	0.294	1.50	0.358	1.56	0.379	1.53	0.364	1.46	0.360	1.49	0.345
	14-16	Male	1.63	0.309	1.53	0.292	1.47	0.341	1.53	0.354	1.35	0.271	1.34	0.254	1.48	0.332	1.49	0.327
		Female	1.36	0.311	1.47	0.250	1.51	0.322	1.48	0.313	1.44	0.335	1.49	0.302	1.46	0.301	1.48	0.322
Externalizing difficulty	11-13	Male	1.45	0.271	1.45	0.315	1.58	0.350	1.58	0.339	1.58	0.342	1.65	0.335	1.55	0.335	1.56	0.336
		Female	1.30	0.266	1.40	0.221	1.48	0.293	1.51	0.286	1.70	0.315	1.61	0.401	1.50	0.326	1.51	0.299
	14-16	Male	1.45	0.172	1.47	0.283	1.55	0.327	1.57	0.341	1.56	0.332	1.53	0.336	1.53	0.329	1.54	0.305
		Female	1.24	0.238	1.43	0.180	1.39	0.302	1.39	0.287	1.43	0.342	1.48	0.353	1.38	0.298	1.42	0.305
Total difficulty	11-13	Male	1.41	0.244	1.44	0.276	1.51	0.266	1.51	0.281	1.46	0.220	1.49	0.209	1.48	0.244	1.49	0.253
		Female	1.28	0.285	1.42	0.176	1.48	0.249	1.50	0.277	1.63	0.258	1.57	0.305	1.48	0.289	1.50	0.260
	14-16	Male	1.54	0.171	1.50	0.193	1.51	0.288	1.55	0.282	1.45	0.243	1.44	0.250	1.50	0.259	1.51	0.261
		Female	1.30	0.240	1.45	0.185	1.45	0.259	1.44	0.247	1.43	0.276	1.48	0.282	1.42	0.253	1.45	0.260
Prosocial behavior	11-13	Male	2.67	0.321	2.62	0.312	2.51	0.393	2.49	0.368	2.75	0.311	2.79	0.288	2.60	0.372	2.59	0.356
		Female	2.71	0.237	2.56	0.274	2.55	0.359	2.57	0.373	2.70	0.347	2.64	0.325	2.61	0.353	2.58	0.363
	14-16	Male	2.64	0.376	2.63	0.450	2.37	0.383	2.33	0.337	2.64	0.298	2.63	0.316	2.49	0.385	2.46	0.384
		Female	2.75	0.248	2.64	0.226	2.59	0.346	2.59	0.362	2.65	0.396	2.68	0.338	2.64	0.336	2.63	0.346
Self-awareness	11-13	Male	2.93	0.398	3.06	0.529	2.90	0.557	2.87	0.553	3.20	0.449	3.12	0.453	2.97	0.537	2.97	0.509
		Female	3.03	0.366	3.01	0.425	2.84	0.521	2.83	0.531	3.02	0.429	3.14	0.523	2.91	0.517	2.92	0.494
	14-16	Male	2.97	0.253	2.98	0.464	2.72	0.636	2.67	0.561	3.12	0.493	3.15	0.483	2.86	0.572	2.85	0.561
		Female	3.39	0.554	3.02	0.440	3.05	0.567	2.96	0.552	3.12	0.521	3.12	0.557	3.14	0.545	3.02	0.551
Self-management	11-13	Male	2.92	0.485	2.85	0.558	2.72	0.534	2.69	0.534	3.09	0.521	2.94	0.586	2.85	0.567	2.78	0.526
		Female	3.11	0.745	2.97	0.414	2.71	0.540	2.74	0.517	2.82	0.534	2.86	0.631	2.80	0.544	2.80	0.581
	14-16	Male	2.89	0.516	2.95	0.691	2.69	0.623	2.70	0.619	3.08	0.391	3.04	0.556	2.82	0.575	2.83	0.632
		Female	3.27	0.498	2.99	0.470	3.01	0.547	2.87	0.553	3.13	0.557	3.10	0.451	3.10	0.514	2.97	0.547
Social awareness	11-13	Male	3.21	0.399	3.25	0.450	2.95	0.631	2.89	0.644	3.47	0.518	3.43	0.511	3.13	0.610	3.09	0.602
		Female	3.39	0.411	3.17	0.368	3.05	0.558	3.06	0.590	3.45	0.395	3.49	0.380	3.19	0.547	3.17	0.529
	14-16	Male	3.32	0.613	3.13	0.616	2.68	0.626	2.66	0.635	3.41	0.392	3.36	0.387	2.98	0.667	2.92	0.654
		Female	3.31	0.489	2.96	0.467	3.11	0.650	3.07	0.638	3.40	0.601	3.38	0.519	3.24	0.584	3.15	0.625
Relationship skills	11-13	Male	3.36	0.532	3.38	0.477	3.07	0.635	3.02	0.567	3.42	0.520	3.36	0.436	3.21	0.541	3.18	0.609
		Female	3.24	0.515	3.24	0.479	2.94	0.570	2.93	0.533	3.06	0.560	3.22	0.531	3.02	0.531	3.05	0.573
	14-16	Male	3.42	0.605	3.28	0.623	2.85	0.564	2.85	0.511	3.36	0.300	3.26	0.438	3.08	0.583	3.03	0.555
		Female	3.32	0.394	3.10	0.563	3.06	0.600	2.99	0.613	3.13	0.499	3.19	0.560	3.13	0.592	3.07	0.539
Responsible decision making	11-13	Male	3.27	0.476	3.34	0.525	3.24	0.498	3.16	0.573	3.43	0.530	3.31	0.526	3.29	0.553	3.24	0.503
		Female	3.43	0.480	3.38	0.410	3.20	0.620	3.08	0.553	3.29	0.416	3.45	0.540	3.26	0.532	3.21	0.581
	14-16	Male	3.33	0.535	3.32	0.550	3.04	0.621	3.09	0.639	3.46	0.439	3.40	0.448	3.20	0.592	3.21	0.594
		Female	3.31	0.448	3.29	0.223	3.37	0.555	3.30	0.598	3.45	0.519	3.47	0.511	3.38	0.518	3.35	0.527
Social emotional learning	11-13	Male	3.14	0.343	3.18	0.397	2.98	0.463	2.93	0.468	3.32	0.368	3.23	0.392	3.09	0.455	3.05	0.437
		Female	3.24	0.345	3.15	0.221	2.95	0.449	2.93	0.429	3.13	0.303	3.23	0.433	3.04	0.416	3.03	0.425
	14-16	Male	3.18	0.307	3.13	0.436	2.80	0.490	2.79	0.491	3.29	0.296	3.24	0.338	2.99	0.472	2.97	0.489
		Female	3.32	0.366	3.07	0.351	3.12	0.474	3.04	0.493	3.25	0.423	3.25	0.400	3.20	0.445	3.11	0.447

For each gender and age-group combination, adolescents report higher internalizing, externalizing, and total difficulties and lower prosocial behavior than perceived by their parents. Moreover, for each gender and age-group combination, adolescents report comparable levels of social emotional learning (self-awareness, self-management, social awareness, relationship skills, responsible decision-making) to those perceived by their parents (see Tables 5, 6). These trends are also visible across countries.

The Kolmogorov-Smirnov test was used to investigate the normality assumption of each scale’s score distribution, and this was carried out separately for each gender, age group, and country. Non-parametric tests were used to analyze the data further, since all the scale distributions were skewed and violated the normality assumption.

For each participant, the changes in their social emotional skills, internalizing/externalizing difficulties, and prosocial scores were generated by subtracting the scores recorded in October 2020 from the scores recorded in May 2021. The Wilcoxon signed-rank test was also used to determine whether the scores differed significantly between the two phases. Table 7 displays the changes in the mean scores or change scores of these eleven scales for each country, gender, and age-group combination (significant changes are marked with an asterisk*).

**TABLE 7:** Adolescent and parent-reported changes in mean SDQ and SSIS-SEL scores and adolescent self-reported CD-RISC-10 scores (n = 512), October 2020 (T1) – May 2021 (T2).

Subscale	Age	Gender	Italy		Latvia		Portugal		Whole Group	
			Child	Parent	Child	Parent	Child	Parent	Child	Parent
Internalizing difficulty	11-13	Male	0.01	0.06	0.06	0.00	0.00	0.00	0.04	0.02
		Female	0.04	0.18	0.06	0.02	0.09	−0.03	0.07	0.03
	14-16	Male	0.13*	−0.10	0.04	0.06	−0.01	−0.01	0.05	0.01
		Female	−0.06	0.11	0.03	−0.03	0.02	0.05	0.01	0.02
Externalizing difficulty	11-13	Male	0.13*	0.00	−0.05	0.00	0.08	0.07	0.02	0.01
		Female	−0.08	0.10	0.08	0.03	0.01	−0.09	0.04	0.01
	14-16	Male	−0.02	0.02	0.05	0.02	0.04	−0.03	0.03	0.01
		Female	−0.03	0.19*	0.07	0.00	−0.06	0.05	0.01	0.04
Total difficulty	11-13	Male	0.07	0.03	0.01	0.00	0.03	0.03	0.03	0.01
		Female	−0.02	0.14*	0.07	0.02	0.05	−0.06	0.05	0.02
	14-16	Male	0.06	−0.04	0.05	0.04	0.02	−0.01	0.04	0.01
		Female	−0.04	0.15*	0.05	−0.01	−0.02	0.05	0.01	0.03
Prosocial behavior	11-13	Male	−0.09	−0.05	−0.01	−0.02	−0.12*	0.04	−0.05	−0.01
		Female	0.04	−0.15*	−0.13*	0.02	0.04	−0.06	−0.06	−0.03
	14-16	Male	−0.25*	−0.01	0.06	−0.04	−0.04	−0.01	−0.02	−0.03
		Female	−0.20*	−0.11	0.02	0.00	0.02	0.03	−0.02	−0.01
Self-awareness	11-13	Male	−0.11	0.13*	0.05	−0.03	−0.07	−0.08	−0.01	0.00
		Female	−0.01	−0.02	−0.11	−0.01	−0.14*	0.12	−0.10	0.01
	14-16	Male	0.02	0.01	0.01	-0.05	-0.02	0.03	0.00	-0.01
		Female	−0.06	−0.37*	−0.08	−0.09	0.00	0.00	-0.05	-0.12
Self-management	11-13	Male	−0.29*	−0.07	−0.07	−0.03	−0.19*	−0.15*	−0.15*	−0.07
		Female	0.16*	−0.14	−0.10	0.03	0.04	0.04	−0.02	0.00
	14-16	Male	−0.11	0.06	0.03	0.01	−0.09	−0.04	−0.02	0.01
		Female	0.19*	−0.28*	0.03	−0.14*	−0.07	−0.03	0.04	−0.13*
Social awareness	11-13	Male	−0.07	0.04	0.04	−0.06	−0.14*	−0.04	−0.02	-0.04
		Female	-0.02	-0.22*	-0.17*	0.01	0.04	0.04	−0.10	−0.02
	14-16	Male	0.20*	−0.19*	0.04	−0.02	0.05	−0.05	0.07	−0.06
		Female	0.10	−0.35*	0.00	−0.04	0.01	−0.02	0.02	−0.09
Relationship skills	11-13	Male	−0.12*	0.02	−0.05	−0.05	−0.04	−0.06	−0.06	−0.03
		Female	0.00	0.00	−0.14*	−0.01	−0.03	0.16*	−0.09	0.03
	14-16	Male	−0.09	−0.14*	-0.06	0.00	0.02	−0.10	−0.04	−0.05
		Female	0.00	−0.22*	−0.13*	−0.07	−0.09	0.06	−0.09	−0.06
Responsible decision-making	11-13	Male	0.04	0.07	0.00	−0.08	−0.04	−0.12*	0.00	−0.05
		Female	−0.21*	−0.05	0.00	−0.12*	−0.03	0.16*	−0.05	−0.05
	14-16	Male	−0.21*	−0.01	0.01	0.05	−0.06	−0.06	−0.05	0.01
		Female	−0.19*	−0.02	0.03	−0.07	−0.04	0.02	−0.03	−0.03
Social emotional learning	11-13	Male	−0.11*	0.04	−0.01	−0.05	−0.09	−0.09	−0.05	−0.04
		Female	−0.02	−0.09	−0.11*	−0.02	−0.02	0.10	−0.08	−0.01
	14-16	Male	−0.03	−0.05	0.01	−0.01	-0.02	−0.05	−0.01	−0.02
		Female	0.01	−0.25*	−0.03	−0.08	−0.04	0.00	−0.02	−0.09
Resilience	11-13	Male	−0.02		−0.06		0.07		−0.02	
		Female	−0.16*		−0.09		0.05		−0.07	
	14-16	Male	−0.48*		−0.08		0.03		−0.14*	
		Female	−0.06		−0.10		0.03		−0.05	

*p < 0.05.

Table 7 shows that some of the changes in the mean internalizing, externalizing, and total difficulty scores and in the mean social emotional learning, resilience, and prosocial behavior scores do not agree in their direction when comparing adolescents’ and parents’ evaluations. Moreover, most of these changes are not significant. This implies that there were few changes in adolescents’ social emotional learning, difficulties, resilience, and prosocial behavior across the whole sample between October 2020 and May 2021. At the same time, several significant changes could be observed in certain age and gender groups when data were analyzed at the country level.

In Italy, adolescents’ self-reports demonstrated a significant decrease in summed social emotional skills, more specifically in self-management and relationship skills among 11-13-year-old boys and responsible decision-making among all 14-16-year-old students and girls aged between 11 and 13. Regarding self-awareness, adolescents’ self-report scores did not show any significant difference, whereas parents reported an increase in this skill in their 11-13-year-old boys and a decrease in 14-16-year-old girls. Contradicting results were also found regarding the social awareness of 14-16-year-old boys, which was self-evaluated as having increased but as having decreased in parents’ reports. Parents also indicated a decreased level of social awareness among both age groups of girls. Parents observed increased total difficulties among girls of all ages, especially externalized difficulties; however, self-report data indicated that externalized difficulties increased among 11-13-year-old boys and internalized difficulties increased among 14-16-year-old boys. Both 11-13-year-old girls and 14-16-year-old boys reported decreased resilience. All students aged 14-16 reported a decrease in their prosocial behavior, which was also observed by parents of 11-13-year-old girls.

In Latvia, all significant changes observed were among girls. In their self-reports, 11-13-year-old girls evaluated a decrease in total social emotional learning, self-awareness, and prosocial behavior. Both 11-13-year-old and 13-16-year-old girls also reported a decrease in relationship skills. Parents observed a decrease in decision-making among 11-13-year-old girls and self-management among 14-16-year-old girls.

In Portugal, according to adolescents’ self-reports, there was a decrease in prosocial behavior, self-management, and social awareness among 11-13-year-old boys and self-awareness among 11-13-year-old girls. Parents also reported decreased self-management among 11-13-year-old boys. Interestingly, parents observed that the dynamic of responsible decision-making was negative for boys and positive for girls in the 11-13-year-old age group. Parents and adolescents only simultaneously perceived a decrease in self-management among 11-13-year-old boys.

Several multilevel models were fitted to relate the difficulties, prosocial behavior, social emotional learning, and resilience scales to three predictors (age, gender, and phase). The aliased terms are age (14-16 years), gender (girl), and phase (October 2020). The model fits included three main effects and two pairwise interaction effects, and these were applied to both the adolescents’ and parents’ evaluations. Table 8 displays the regression coefficients, standard errors, and p-values of each model. We fitted appropriate models to investigate how these change scores vary between phase (2020, 2021), gender (boys, girls) and age (11-13, 14-16).

**TABLE 8:** Regression coefficients, standard errors, and p-values of the models for SSIS-SEL, SDQ, and CD-RISC-10 with age, gender, and phase as predictors.

	Adolescents			Parents		
	B	SE	*P*-value	B	SE	*P*-value
**Internalizing difficulty**						
Age (11-13 years)	−0.075	0.032	0.018	−0.035	0.029	0.223
Gender (Boy)	−0.155	0.032	< 0.001	−0.018	0.029	0.540
Phase (May 2021)	0.025	0.038	0.512	0.009	0.036	0.804
Gender (Boy) * Phase (May 2021)	0.004	0.045	0.930	0.018	0.040	0.656
Age (11-13 years) * Phase (May 2021)	0.024	0.045	0.585	0.007	0.040	0.862
**Externalizing difficulty**						
Age (11-13 years)	−0.028	0.028	0.319	−0.073	0.028	0.010
Gender (Boy)	0.040	0.028	0.155	0.101	0.028	< 0.001
Phase (May 2021)	0.021	0.034	0.523	0.007	0.034	0.839
Gender (Boy) * Phase (May 2021)	0.001	0.040	0.985	0.016	0.040	0.688
Age (11-13 years) * Phase (May 2021)	0.008	0.040	0.849	0.016	0.040	0.687
**Total difficulty**						
Age (11-13 years)	−0.051	0.025	0.039	−0.014	0.023	0.532
Gender (Boy)	0.058	0.025	0.021	0.025	0.023	0.284
Phase (May 2021)	0.023	0.030	0.437	0.033	0.027	0.230
Gender (Boy) * Phase (May 2021)	0.002	0.035	0.947	0.017	0.033	0.603
Age (11-13 years) * Phase (May 2021)	0.016	0.035	0.650	0.004	0.033	0.891
**Prosocial behavior**						
Age (11-13 years)	0.052	0.031	0.097	0.041	0.032	0.207
Gender (Boy)	−0.125	0.031	< 0.001	−0.082	0.032	0.011
Phase (May 2021)	−0.019	0.039	0.634	−0.015	0.040	0.705
Gender (Boy) * Phase (May 2021)	0.004	0.044	0.922	0.003	0.045	0.946
Age (11-13 years) * Phase (May 2021)	−0.036	0.044	0.409	−0.003	0.045	0.951
**Social emotional learning**						
Age (11-13 years)	0.054	0.032	0.094	0.035	0.040	0.384
Gender (Boy)	−0.152	0.032	< 0.001	−0.078	0.040	0.052
Phase (May 2021)	−0.006	0.041	0.881	−0.015	0.049	0.765
Gender (Boy) * Phase (May 2021)	0.019	0.046	0.684	0.015	0.056	0.793
Age (11-13 years) * Phase (May 2021)	0.047	0.046	0.304	0.034	0.056	0.543
**Resilience**						
Age (11-13 years)	0.039	0.062	0.534			
Gender (Boy)	0.228	0.062	< 0.001			
Phase (May 2021)	−0.034	0.076	0.654			
Gender (Boy) * Phase (May 2021)	−0.019	0.088	0.831			
Age (11-13 years) * Phase (May 2021)	0.046	0.088	0.602			

*refers to the interaction of two explanatory variables.

The main effect of age and gender reached statistical significance; however, inconsistent results were obtained from parents’ and adolescents’ reports. Adolescents, but not their parents, agree that, on average, boys score lower than girls and that adolescents aged 11-13 years score lower than their older counterparts on internalizing difficulties. At the same time, parents, but not adolescents themselves, evaluated boys’ scores higher than girls and younger adolescents lower than older ones on externalizing difficulties.

Both sets of informants agreed that adolescent boys score lower than girls on prosocial behavior and social emotional learning. Boys demonstrated higher resilience scores compared to girls.

The main effect of the phase was not significant, demonstrating that, on average, changes between October 2020 and May 2021 were not significant when individual factors (age and gender) were controlled.

No pairwise interaction effects were significant either, indicating that no particular age or gender groups were identified in the sample as a whole as being more affected by the pandemic experience.

Multilevel models are hierarchical linear mixed models that facilitate the analysis of hierarchical data particularly when observations are nested within higher levels of classification. These models are extensions of regression models and accommodate well the levels of our clustered data set in which individuals (adolescents or parents) are nested within school levels (lower and higher secondary) and schools are nested within countries (Italy, Latvia, and Portugal). Tables 9, 10 display the variances and intraclass correlations at each level of nesting for the multilevel models fitted to the difficulty, prosocial, resilience and SEL subscale scores. The STATA GLLAMM routine was used to fit these multilevel models.

**TABLE 9:** Variances and Intraclass correlations at levels 1, 2 and 3 (Adolescent Evaluations).

Dependent Variable	Individual Level 1		School Level 2		Country Level 3	
	Variance	Intraclass correlation	Variance	Intraclass correlation	Variance	Intraclass correlation
Internalizing Difficulty	0.2562	0.9599	0.0075	0.0281	0.0032	0.0120
Externalizing Difficulty	0.2308	0.8674	0.0136	0.0511	0.0217	0.0815
Total Difficulty	0.2220	0.9254	0.0064	0.0267	0.0115	0.0479
Prosocial	0.2448	0.9241	0.0093	0.0351	0.0108	0.0408
Resilience	0.4121	0.9324	0.0153	0.0346	0.0146	0.0330
Self-Awareness	0.2968	0.9416	0.0064	0.0203	0.0120	0.0381
Self-Management	0.3393	0.9089	0.0084	0.0225	0.0256	0.0686
Social Awareness	0.4031	0.9126	0.0097	0.0220	0.0289	0.0654
Relationship Skills	0.3113	0.8894	0.0072	0.0206	0.0315	0.0900
Responsible Decision Making	0.3415	0.9232	0.0088	0.0238	0.0196	0.0530
Social Emotional Learning	0.3005	0.9530	0.0046	0.0146	0.0102	0.0324

**TABLE 10:** Variances and Intraclass correlations at levels 1, 2 and 3 (Parent Evaluations).

Dependent Variable	Individual Level 1		School Level 2		Country Level 3	
	Variance	Intraclass correlation	Variance	Intraclass correlation	Variance	Intraclass correlation
Internalizing Difficulty	0.2521	0.9553	0.0063	0.0239	0.0055	0.0208
Externalizing Difficulty	0.2597	0.9654	0.0042	0.0156	0.0051	0.0190
Total Difficulty	0.2487	0.9580	0.0033	0.0127	0.0076	0.0293
Prosocial	0.2349	0.9767	0.0014	0.0058	0.0042	0.0175
Self-Awareness	0.3593	0.9721	0.0024	0.0065	0.0079	0.0214
Self-Management	0.4085	0.9582	0.0052	0.0122	0.0126	0.0296
Social Awareness	0.4163	0.9581	0.0049	0.0113	0.0133	0.0306
Relationship Skills	0.3671	0.9567	0.0049	0.0128	0.0117	0.0305
Responsible Decision Making	0.3885	0.9597	0.0065	0.0161	0.0098	0.0242
Social Emotional Learning	0.2721	0.9511	0.0074	0.0258	0.0066	0.0231

For the parent and student evaluations, the intra cluster correlations at individual level ranged from 0.8674 to 0.9767; the intra cluster correlations at school level ranged from 0.0113 to 0.0511; while the intra cluster correlations at country level ranged from 0.0120 to 0.0900.

### Relationship of social emotional learning to adolescents’ resilience and behavioral problems during the COVID-19 pandemic

Using the adolescents’ evaluations, the Spearman correlation test using change scores between T1 and T2 was used to measure the strength of the relationships between changes in social emotional learning scores and changes in difficulty, prosocial behavior, and resilience scores. Table 11 shows that for each country, gender, and age-group combination, changes in social emotional learning scores are negatively related to changes in difficulty scores but positively related to changes in prosocial behavior and resilience scores. Moreover, most of these relationships are significant at the 0.05 level of significance. This implies that the adolescents who had the largest reductions in their social emotional learning scores between October 2020 and May 2021 had the largest reductions in their prosocial behavior and resilience scores and had the largest increments in their internalizing and externalizing scores. On the other hand, adolescents who experienced a greater development of their social emotional learning skills also experienced a larger increase in resilience and prosocial behavior and a decrease in difficulties.

**TABLE 11:** Correlation between changes from T1 to T2 in SSIS-SEL and changes in SDQ and CD-RISC-10.

Subscale	Age	Gender	Italy	Latvia	Portugal	Whole Group
Internalizing difficulty	11-13	Male	−0.315*	−0.147	−0.158	−0.186
		Female	−0.185	−0.271*	−0.339*	−0.273*
	14-16	Male	−0.126	−0.129	−0.130	−0.117
		Female	−0.138	−0.188	−0.355*	−0.232*
Externalizing difficulty	11-13	Male	−0.133	−0.227*	−0.287*	−0.222*
		Female	−0.196	−0.348*	−0.358*	−0.330*
	14-16	Male	−0.223	−0.518*	−0.285*	−0.344*
		Female	−0.428*	−0.285*	−0.167	−0.252*
Total difficulty	11-13	Male	−0.232*	−0.159	−0.273*	−0.207*
		Female	−0.189	−0.369*	−0.390*	−0.353*
	14-16	Male	−0.192	−0.351*	−0.267*	−0.293*
		Female	−0.364*	−0.249*	−0.318*	−0.289*
Prosocial behavior	11-13	Male	0.320*	0.407*	0.631*	0.482*
		Female	0.523*	0.554*	0.380*	0.476*
	14-16	Male	0.430*	0.320*	0.259*	0.278*
		Female	0.303*	0.377*	0.294*	0.318*
Resilience	11-13	Male	0.386*	0.270*	0.611*	0.464*
		Female	0.463*	0.273*	0.306*	0.372*
	14-16	Male	0.442*	0.228*	0.293*	0.342*
		Female	0.466*	0.239*	0.396*	0.374*

*p < 0.05.

## Discussion

### Changes in social emotional skills, resilience, and difficulties among adolescents during the pandemic

The first aim of the current study was to explore the changes in social emotional skills, resilience, and behavioral problems among different age and gender groups of adolescents in three European countries during the COVID-19 pandemic using data from both adolescents’ self-reports and their parents’ reports.

Addressing changes in adolescents’ *social emotional skills*, most of them did not increase, and some of them even demonstrated a decrease. It is to be expected that social emotional skills should increase following normative developmental trajectories [e.g., (4, 5)]. However, a significant decrease in adolescents’ self-management skills was reported by 11-13-year-old boys and 14-16-year-old girls’ parents. This finding demonstrate that the pandemic experience has raised significant obstacles to the healthy development of the adolescents, given that social emotional learning is a crucial component of their social maturity.

This can be explained by the fact that educational practices during the pandemic were affected to a large extent in many countries. It was estimated that the school routine changed rapidly, starting from the beginning of the pandemic, and did not provide the necessary support for social emotional learning. Remote learning was used as an alternative to traditional education practice but was implemented with great variety and encountered several obstacles and challenges. First, the necessity to switch to remote learning was demanding for all involved in the education process – students, teachers, and parents [e.g., (37, 69)]. Remote learning was a challenge for the self-management skills of early adolescents. At the same time, it can be assumed that certain social emotional skills received more attention (e.g., self-management and responsible decision-making) because they became salient, especially during self-guided or independent learning, and parents had more opportunities to observe them because both adolescents and parents spent more time at home. Second, during this stage of the pandemic, schools were not a supportive environment for social emotional learning. Even before the pandemic, social emotional learning was often not perceived as a routine in the education curricula, and therefore it was not perceived as a priority within other fields (e.g., mathematics) in situations when teachers were faced with increased demands and stress. Third, restrictions related to social distancing decreased interaction possibilities both at school and in extra-curricular activities, limiting necessary space for practicing relationship skills. The aforementioned could be perceived as challenging for a normative development during adolescence [e.g., (6–9)]. Even those adolescents with no previous vulnerabilities were faced with situation, when optimal course of development of their identity, healthy separation from families and skills of social communication was jeopardized. From the one side, the COVID-19 pandemic requested more strong social emotional skills to overcome challenges, but from the other side, it worked as a mitigating force against the social emotional learning.

Addressing changes in adolescents’ *resilience*, no positive dynamic was observed. This can be explained by the necessity to grow through difficulties to develop resilience. By T2, there had probably not yet been sufficient time for the growth of the experience of the pandemic to reach its potential. On the contrary, resilience decreased among 14-16-year-old boys, and the decrease was more notably expressed in the Italian sample. Comparing these results with other resilience measures obtained to date, it is known that social emotional skills are positively related to resilience [e.g., (37)]. If social emotional skills decreased between the first and second measure points of this research, then it is plausible that resilience also decreased. The decrease in adolescent boys’ resilience observed in the Italy sample provides insights that negative dynamics can be observed, and research on resilience changes during pandemics should be continued.

Addressing *social, emotional, and behavioral difficulties*, it was found that both internalized and externalized difficulties showed a tendency to increase and prosocial behavior to decrease; however, none of them across the whole sample reached a level of statistical significance.

This finding does not replicate results from previous studies [e.g., (15)] but is in line with results from one previous study (31). Studies demonstrating the pandemic’s effect on adolescents have mostly used clinical indicators such as depression and anxiety. It can be assumed that the SDQ measures used in this study, albeit useful for screening purposes, were not sensitive enough to estimate potential threats to the mental health of adolescents during the pandemic. For example, Bignardi and colleagues (45) found weak, non-significant changes in the SDQ emotional difficulties scale but a medium to large increase in depression (measured with the Revised Child Anxiety and Depression Scale). Another explanation could be linked to the longitudinal design and timing of the current study. Significant changes can be observed when baseline rates are compared with those observed after the short period of pandemic exposure. At the same time, the mental health indicators can be observed to fluctuate in longitudinal studies with several repeated measures over a longer period. For example, in their 12-month study, Shum and colleagues (28) observed an increase in mental health issue symptoms when restrictions were at their highest as well as a decrease when restrictions eased. In light of this finding, we can speculate that even if there was a fluctuation between T1 and T2 with the peak rates around the time of the strongest restrictions, it was followed by a decrease related to loosened restrictions in spring 2021 at the time of the T2 measure. This allows us to assume that the pandemic influenced certain vulnerable groups who experienced rapid damage to their mental health (also observed by an increased need for psychiatric care) and had an immediate effect on them, but there is also the issue of adaptation.

The multilevel models demonstrate that the individual level-1 variance explains more than 90% of the total variation in the data, which implies that changes in adolescents’ social emotional learning, prosocial behavior, difficulties and resilience varied more between individuals than between schools and countries. No age or gender group was more affected than the others when considering changes during the pandemic in social emotional learning, resilience, and internalized and externalized difficulties. However, average non-significant changes over the whole sample and contradictory findings within different age and gender groups cover huge individual differences and variety, assuming that specific groups could be considered as more vulnerable when facing pandemic-related difficulties. At the same time, several significant changes were observed in certain age and gender groups when data were analyzed at a country level, and most of the significant changes were observed in the Italian sample. This highlights the necessity to analyze environmental factors to explain the country-level differences, considering that the epidemiological situation was different in Italy, Latvia, and Portugal during this period of the pandemic, as were the restriction policies and adaptation of the learning environment.

#### Italy

Italy was characterized by the most intense pandemic experience compared to Portugal and Latvia. It was exposed to the pandemic intensively from the beginning of 2020 and entered this study with higher prevalence and cumulative death rates, characterizing prolonged exposure to the stress of the global pandemic.

There were more significant changes in Italy compared with other countries considering social emotional learning, resilience, and behavioral difficulties and more diverse results. For instance, resilience was only reported as having decreased by adolescents. However, if we consider the total Italian scores for social emotional skills and prosocial behavior in both informant reports, the scores have mostly the same trend, which is a decrease; emotional and behavioral difficulties tended, instead, to increase, as found in previous studies (1, 13, 15, 40). It could be explained that because social emotional learning decreased, resilience decreased as well due to their strong and positive association [e.g., (37)], and total difficulties increased in turn. Students were exposed to prolonged social isolation and intense negative feelings and experiences, which may explain lower scores in social emotional learning and resilience at T2. The result that prosocial behaviors decreased emphasizes the actual situation during the pandemic when adolescents did not have many opportunities to demonstrate their prosocial skills. Indeed, students in the 11-16-year-old age range were heavily penalized during the pandemic as restrictions were strict for them across the whole school year. Italian adolescents used to go out with friends, do group sports activities, and have many social opportunities to be with peers. Prolonged distance learning, social distancing, and the closure of many leisure/sports centers represented a loss of opportunities to meet peers, which may have been detrimental to the development and practice of both social emotional skills and prosocial behavior.

#### Latvia

There were low prevalence and cumulative death rates in Latvia at the beginning of this study in October 2020. However, there had been a rapid increase in both prevalence and mortality rates by May 2021, demonstrating growing and consistently high exposure to the stress of the pandemic and epidemiological measures. During the current study, most participants experienced a period of remote learning (from 4 to 7 months). Remote learning was characterized by an increased proportion of independent tasks, a decreased number of topics, and a great variety of interaction opportunities, and it was evaluated by parents in Latvia as a significant source of stress (69, 70).

Differences found in the Latvian sample were gender asymmetric, and all significant changes observed were exclusively among girls. This is in line with findings from other studies (2, 14, 16) indicating that girls are more affected by the adverse experience of the pandemic. An explanation for this could be related to environmental factors. There is evidence that the educational environment in Latvia was more supportive for girls than boys, including important gender disparities in terms of the level of attainment up to tertiary education (71). It can be assumed that rapid changes in educational routine probably affected girls to a larger extent than boys. Another possible explanation is related to available resources. Extracurricular activities have been recognized as a significant resource for the development and well-being of adolescents (72); however, the observed effect is stronger for boys. During the period of restrictions during the pandemic, outdoor group sports activities were allowed in Latvia, but art, dance, and music classes (taking place indoors) were eliminated. Traditionally, more boys are involved in sports activities than girls, resulting in gender asymmetry in available resources for successful coping mechanisms and healthy development (communications, support, and physical activity). This could be assumed as one possible explanation for gender differences in changes observed during the pandemic in the Latvian sample. Nevertheless, the protective role of extra-curricular activities especially for boys would be as a direction for further research.

A significant protective factor that could be assumed in the Latvian sample is related to the availability of outdoor space, as most of the adolescents involved in the study live in small towns or in the countryside where there is a low population density. Outdoor leisure activities such as cycling, walking, hiking, and gardening were available to them, providing a valuable resource for maintaining physical and mental well-being (73). However, these activities are more characteristic for boys than girls.

#### Portugal

Portugal was characterized by a moderately high exposure to the pandemic at the beginning of the study in October 2020 and by a rapid increase and then a significant decrease in its prevalence, albeit with high death rates.

As far as the results obtained in the Portuguese sample are concerned, it can be assumed that most adolescents were able to maintain their social and emotional competencies through the pandemic, as mentioned in other studies (2, 74). Such results may also be related to protective factors related to the various school measures adopted in Portugal, which allowed more flexible and less distracting learning arrangements and self-paced and independent learning models, enabling the development of individual agency, self-advocacy, and time-management skills (75).

Despite this, the results show a worsening of social emotional learning among younger boys, which can be explained by an increased vulnerability of boys that is related to the later development of such competencies in boys compared with girls (76).

In sum, these results are in line with previous findings. A systematic review (2) reported that 93% of children could cope with lockdown measures. However, it also highlighted the need to recognize protective factors and resources.

### The protective role of social emotional learning

The second aim of our study was to explore the relationship between social emotional learning and adolescents’ resilience and behavioral problems during the COVID-19 pandemic.

Results both across the whole sample and at a country level demonstrate a clear pattern – changes in social emotional learning were related to changes in internalized and externalized difficulties. That means that those adolescents who experienced a larger increase in social emotional learning were better protected from the possible adverse effects of pandemic-related stress and vice versa – those students who had not succeeded in acquiring social emotional skills were faced with larger threats to their mental health and resilience development. Correlation coefficients ranged from weak to moderate, demonstrating that such skills as self-management, self-awareness, empathy, relationship skills, and responsible decision-making play a protective role when facing a global source of stress at an adolescent age.

This finding contributes to previous evidence supporting the protective and resource role of social emotional learning on the resilience of adolescents (37) and academic outcomes, even at a preschool level (77).

Our findings are in line with those observed in a study in Finland (31), where it was found that the majority of adolescents demonstrated no changes in social emotional skills; however, those who experienced growth also reported more stable or even increased academic well-being (in terms of engagement and chance of burnout).

The current study demonstrates the necessity to pay special attention to social emotional learning in the context of the global stress of the pandemic, as it has a protective role against emotional and behavioral difficulties and a promotional effect on resilience development. The “Promoting mental health at schools” (PROMEHS) program, including a ready-to-use activity plans for children and adolescents, fostering their social emotional learning and resilience, and mitigating development of behavioral problems, was designed to be provided in existing contexts without a pandemic. However, it proved to be a protective factor for both students and their teachers during the time of the COVID-19 pandemic (78, 79), thus contributing to the protection of mental health during this extreme situation.

## Conclusions and implications

It can be concluded that, in the general sample, we observed minor changes in adolescents’ social emotional learning and resilience and no significant changes in their internalized and externalized difficulties. At the same time, it can be concluded that, in specific age and gender groups, there are significant differences, demonstrating the large variability of the pandemic’s effect and the need for an individualized perspective emphasizing age and gender differences. This study provides evidence that prolonged intensive exposure to the pandemic can have a more significant effect on adolescents’ mental health than in case of a less prolonged or intensive experience of the pandemic. Our findings confirm the protective role of social emotional learning, even in the context of the global stress related to the COVID-19 pandemic. Some contradictory results demonstrate the huge individual differences and diversity involved when adapting to pandemic-related stress and restrictions.

The practical implications emphasize the necessity to recognize individuals and groups for whom social emotional learning is difficult and to make an effort to strengthen them through training and support. Another practical implication is related to schools – social emotional learning should be prioritized during times of adversity and when considering limitations of resources.

## Strengths and limitations

This study emphasizes the significance of longitudinal, multi-informant, cross-country research on the dynamic of adolescent social emotional learning, resilience, and behavioral problems during the COVID-19 pandemic. The three countries involved in the study experienced different dynamics during the pandemic and different epidemiological situations at the beginning of the study. However, the situation was comparable in terms of the restrictions and learning routines implemented in each country, providing solid ground for cross-country comparisons.

The limitations of the study are related to the reliability of the self-report form of SDQ and SSIS-SEL. However, reliability estimates in the current study are comparable with those obtained in previous research. Another limitation may be associated with the selection of the sample, which was based on convenience sampling; however, the recruitment strategy was population-based, although the initial response rate was high (e.g., 95% in Latvia), the rate of excluded cases, where only one informant (either the adolescent or the parent) answered the questionnaire, was considerable in all participant countries.

In this study, variables such as presence of a family conflict or availability of social support, known as having impact on emotional development and behavioral difficulties of adolescents were not measured. These aspect must be considered when drawing conclusions about consequences of the pandemic on adolescents’ mental health.

## Data availability statement

The raw data supporting the conclusions of this article will be made available by the authors, without undue reservation.

## Ethics statement

The studies involving human participants were reviewed and approved by the Ethics Committee for Humanities and Social Sciences Research Involving Human Participants of the University of Latvia. The Ethics Committee of the Environmental Health Institute at the University of Lisbon. The Ethical Committee of the University of Milano-Bicocca. Written informed consent to participate in this study was provided by the participants’ legal guardian/next of kin.

## Author contributions

BM: lead writer, arranging the research in Latvia, and collecting data. ISt: contributing to writing. ID and IS: arranging the research in Latvia and collecting data. CS: arranging the research in Portugal and contributing to writing. EC: a key contribution in arranging the research in Italy, collecting data, and writing and revising the manuscript. VC: contributing to designing the research, arranging the research in Italy, and collecting data. AA and SG: arranging the research in Italy and collecting data. IG and VO: a key contribution to designing the research and revising the manuscript. LC: data analyses, contributing to writing. All authors contributed to the article and approved the submitted version.
